# Dietary sophorolipid accelerates growth by modulation of gut microbiota population and intestinal environments in broiler chickens

**DOI:** 10.1186/s40104-021-00606-x

**Published:** 2021-07-12

**Authors:** Min-Jin Kwak, Min-Young Park, Yong-Soon Choi, Junghwan Cho, Duleepa Pathiraja, Jonggun Kim, Hanbae Lee, In-Geol Choi, Kwang-Youn Whang

**Affiliations:** 1 Department of Biotechnology, Korea University, 145 Anam-ro, Seoul, 02841 Republic of Korea; 2 Pathway Intermediates, Seoul, 02841 Republic of Korea

**Keywords:** Broilers, Gut microbiota, Gut morphology, Local inflammation, Mucus barrier, Sophorolipids

## Abstract

**Background:**

Gut is a crucial organ for the host’s defense system due to its filtering action of the intestinal membrane from hazardous foreign substances. One strategy to strengthen the gut epithelial barrier function is to upregulate beneficial microflora populations and their metabolites. Sophorolipid (SPL), which is a glycolipid bio-surfactant, could increase beneficial microflora and decrease pathogenic bacteria in the gastrointestinal tract. Therefore, herein, we conducted an experiment with broiler chickens to investigate the fortifying effects of SPL on the host’s gut defense system by modulating the microbiota population.

**Methods:**

A total of 540 1-day-old chicks (Ross 308) were used, and they were immediately allotted into three treatment groups (6 replications with 30 chicks/pen) according to their initial body weight. The dietary treatments consisted of CON (basal diet), BAM (10 mg/kg bambermycin), and SPL (10 mg/kg SPL). During the experiment, birds freely accessed feed and water, and body weight and feed intake were measured at the end of each phase. On d 35, birds (one bird/pen) were sacrificed to collect jejunum and cecum samples.

**Results:**

Dietary SPL and BAM supplementation significantly accelerated birds’ growth and also significantly improved feed efficiency compared to CON. Intestinal microbial community was significantly separated by dietary SPL supplementation from that of CON, and dietary SPL supplementation significantly increased *Lactobacillus* spp. and *Akkermansia muciniphila*. Moreover, birds fed with dietary SPL also showed the highest concentration of cecal butyrate among all treatment groups. Gut morphological analysis showed that dietary SPL significantly increased villus height, ratio of villus height to crypt depth, goblet cell numbers, and the gene expression levels of claudin-1 and mucin 2. Additionally, dietary SPL significantly decreased the mRNA expression level of pro-inflammatory cytokine, interleukin-6, and increased that of anti-inflammatory cytokine, interleukin-10, compared to other treatments.

**Conclusions:**

Dietary SPL increases the beneficial bacterial population and butyrate concentration, which leads to a strengthened gut barrier function. In addition, the intestinal inflammation was also downregulated by dietary SPL supplementation.

## Introduction

In the livestock industry, antibiotics have been widely used as growth promoters with sub-therapeutic dosage due to their outstanding efficacy in feed conversion and animal growth [1]. Nonetheless, the use of antibiotic growth promoters has been banned because the livestock fed with antibiotic growth promoter could serve as a reservoir of antibiotic-resistant bacteria [2]. Hence, it could threaten human life by transmitting antibiotic-resistant bacteria to humans by direct animal contact or indirect environmental contact [3].

Consequently, the development of novel and eco-friendly materials (e.g., probiotics, prebiotics, organic acids, essential oils, and enzymes) to replace antibiotic growth promoters is needed [4]. Additionally, various bio-surfactants have been investigated because of their antibacterial property [5]. Among bio-surfactants, sophorolipid (SPL) has received much attention in various industrial fields, such as medical, hygiene, and pharmaco-dermatological areas, due to their relatively less toxicity and more biodegradability [6].

SPL is a glycolipid-type amphiphilic compound similar to bambermycin, and it is produced by non-pathogenic yeast species, including *Candida bombicola* [7]. It consists of a nonpolar fatty acid tail of 16 or 18 carbon atoms and a polar dimetric carbohydrate head, which is linked by a glycosidic bond and binding of non-polar fatty acid and polar carbohydrate determines structure of SPL, acidic and lactonic forms [8]. Both forms of SPL are able to be produced by *Candida bombicola*, and they exert diverse biological properties according to their forms [9]. In acidic form, SPL shows high solubility with foam-forming ability by its higher esterification capacity, however, lactonic form of SPL exhibits better surface tension reducing activity and biological activity [10].

Collectively, SPL exerts diverse biological properties, including antibacterial activity, immunomodulation capacity, and stimulation of dermal fibroblasts and collagen production, [11, 12]. These properties imply that SPL has a potential to be applied in the animal feed industry for improving animal health and growth however, there are few reports on the application of this compound in this field. Hence, we conducted an experiment to evaluate the efficacy of SPL on gut microbial population and their metabolites could lead to improvement of growth performance in in broiler chickens.

## Materials and methods

We conducted all of the studies on the birds in accordance with the guidelines and regulations of the Animal Ethics Committee approved by Korea University (Seoul, Republic of Korea), and it was carried out at a research farm in Cheonan, Republic of Korea (Approval number: KUIACUC-2020-0097).

### Birds and diets

A total of 540 1-day-old male chicks (Ross 308) were used, and they were allotted into three experimental treatment groups according to their body weight (BW; initial BW: 40.1 g). Each treatment had six replicates with 30 chicks per pen. The dietary treatments consisted of CON (basal diet), BAM (10 mg/kg of bambermycin-supplemented diet), and SPL (10 mg/kg SPL supplemented diet). The feed composition is shown in Table 1, and the feed and SPL were supported by EASY BIO Inc. (Seoul, Republic of Korea). Birds freely accessed feed and water during the experiment, and their BW and feed intake were measured at the end of each phase (Phase 1: d 0–10; phase 2: d 11–20; phase 3: d 21–35) after 8 h of feed deprivation to calculate average daily gain (ADG), average daily feed intake (ADFI), and feed efficiency (FE). Birds were raised in a controlled experimental room with a rice hull with an average relative humidity of 60%. Temperature was maintained for 3 d at 30 °C and daily reduced by 0.5 °C to 24 °C, and lightening was provided by artificial light for 24 h/d.

**Table 1:** Ingredients and nutritional values of basal diets

Ingredients, %	Starter	Grower	Finisher
Corn	60.92	56.29	62.07
Soybean meal	26.53	18.25	10.63
Fermented soybean meal	5.00	0.00	0.00
DDGS	0.00	5.00	5.00
Unpolished rice	0.00	4.00	3.00
Rice bran polish	0.00	1.00	1.50
Rapeseed mineral	0.00	4.00	3.00
Sesameseed meal	0.00	0.00	0.50
Poultry meal	2.50	5.50	8.00
Animal fat	0.00	1.72	2.00
L-Lysine sulfate (55%)	0.62	0.72	0.75
L-Methionine (90%)	0.47	0.35	0.31
Threonine (98%)	0.22	0.19	0.19
L-Tryptophan (99%)	0.01	0.03	0.03
Choline chloride (50%)	0.10	0.11	0.14
MCP	1.54	1.08	0.81
Limestone	1.45	1.23	1.55
Salt	0.25	0.25	0.25
NaHCO_3_	0.05	0.05	0.05
Vitamin premix^a^	0.20	0.14	0.11
Mineral premix^b^	0.15	0.12	0.12
Total	100.00	100.00	100.00
Calculated value			
ME, kcal/kg	2851.00	2945.00	3040.00
CP, %	21.85	20.40	19.00
Ca, %	1.00	0.90	1.04
P, %	0.77	0.70	0.64
Lysine, %	1.49	1.32	1.19
Methionine, %	0.75	0.62	0.56
Threonine, %	1.02	0.94	0.89
Tryptophan, %	0.25	0.23	0.20

^a^Provided per kilogram of complete diet: vitamin A, 6300 IU; vitamin D, 2,800 IU; vitamin E, 35 mg; vitamin K_3_, 1.75 mg; vitamin B_1_, 2 mg; vitamin B_2_, 6 mg; vitamin B_6_, 3 mg; vitamin B_12_, 13 μg; biotin, 0.1 mg; calcium pantothenic acid, 15 mg; folic acid, 1.5 mg; niacin, 50 mg

^b^Provided per kilogram of complete diet: Mn, 100 mg; Cu, 17 mg; Zn, 92 mg; Fe, 50 mg; I, 1.5 mg; Co, 0.15 mg; Se, 0.3 mg

### Sample processing

At the end of the experiment, 18 birds (one bird per pen, randomly selected) were sacrificed, and the weight and length of the small intestine of the birds were measured. Jejunum and cecum samples were collected, immediately frozen, and stored at − 80 °C until further analysis. Parts of jejunum samples were fixed in 4% formalin solution for histological analysis.

### Next generation sequencing

Quick-DNA™ Fecal/Soil Microbe Microprep Kit (Zymo Research, CA, USA) was used to extract total genomic DNA from the cecal samples of chicks according to the manufacturer’s protocol. For the microbial community structure analysis, V3-V4 regions of 16S rDNA were amplified using the following universal primer set (forward 5′-CCTACGGGNGGCWGCAG-3′, reverse 5′- GACTACHVGGGTATCTAATCC-3′), synthesized by Integrated DNA Technologies (IDT, Singapore). PCR conditions for amplifying the V3-V4 regions of 16S rDNA were as follows; 5-min initial denaturation at 98 °C, followed by 20 amplification cycles (30 s at 98 °C, 30 s at 56 °C, 1 min 30 s at 72 °C), 5 min final extension at 72 °C. The resulting DNA amplicons were purified using magnetic beads (TopQ XSEP MagBead, CELLMICS, USA). A second PCR was performed to attach the Illumina universal p5/p7 overhang sequence and sample-specific barcodes. Second PCR conditions were as follows; 1-min initial denaturation at 98 °C, followed by 10 amplification cycles (30 s at 98 °C, 30 s at 60 °C, 1 min at 72 °C), final extension at 72 °C 3 min. The sequencing-ready libraries were purified using magnetic beads (TopQ XSEP MagBead, CELLMICS, USA). Size distribution of the sequencing ready libraries were determined using the QSep fragment analyzer (Qsep Inc., Taiwan). DNA quantification is performed using the Quit dsDNA HS assay kit (Thermo Scientific, USA). Finally, all the libraries were pooled in equimolar quantities and diluted as necessary. Sequencing was performed on the Illumina MiSeq platform (Illumina, USA) using MiSeq Reagent Kit V3 (2 × 300 PE) (Illumina, USA). Mock DNA libraries were prepared with the ZymoBiomics microbial community DNA standard (Zymo Research, USA) using the amplicon library preparation procedure described above and sequenced together. This control library allowed us to evaluate potential biases and errors associated with the amplification and sequencing steps. Taxonomy profiling was performed using the open software program Quantitative Insights Into Microbial Ecology (QIIME) version 1.9.0 with NCBI 16S DB and BLASTN v2.3.0 [13]. We also performed a closed-reference operational taxonomic unit picking process to assign taxonomy using NCBI 16S DB and BLASTN (v2.3.0).

### Gas chromatography–mass spectrometry

The concentration of short-chain fatty acids (SCFA) in the cecum contents was determined by gas chromatography–mass spectrometry (GC***–***MS). Briefly, 10 mg of cecal contents were homogenized with extraction solution consisting of 100 μL of internal standard (100 μmol/L crotonic acid), 100 μL hydrochloric acid, and 200 μL of ether. After vigorous vortexing for 10 min, the homogenates were centrifuged at 1,000×*g* for 10 min, and 80 μL of supernatants were transferred into new glass vials. Aliquots were mixed with 16 μL of *N*-tert-butyldimethylsilyl-*N*-methyltrifluoroacetamide (MTBSTFA) and sealed tightly. The glass vials were heated at 80 °C for 20 min in a water bath, and then left at room temperature for 48 h for derivatization. The derivatized samples were run through a 6890 N Network GC System with an HP-5MS column and 5973 N network mass selective detector. Pure helium was used as carrier gas and delivered at a 1.2 mL/min flow rate. The head pressure was set at 97 kPa with a 20:1 split. The inlet temperature was 250 °C, and the transfer line temperature was 260 °C. The temperature program was as follows: 60 °C for 3 min, 60–120 °C (5 °C per min), and 120–300 °C (20 °C per min). The run time was 30 min, and SCFA concentrations were quantified by comparing their peak areas with standards.

### Gut histological assay

Jejunum samples, fixed in formalin, were embedded into paraffin blocks to prepare 5-μm cross sections using a Rotary Microtome CUT 5062 (SLEE MAINZ, Mainz, Germany). These sections were stained with hematoxylin and eosin and Alican blue staining methods. Total 10 villi and 10 crypts were randomly selected per experimental unit. A single observer measured villus height (VH) and crypt depth (CD) and counted the goblet cells numbers.

### qRT-PCR analysis

Total RNA from jejunum samples was extracted using Trizol® (Invitrogen, Grand Island, NY, USA) according to the manufacturer’s procedure, and the concentration and purity of RNA were determined using Nanodrop spectrophotometer (Thermo Scientific, Wilmington, DE, USA). Subsequently, cDNA samples were synthesized with the High-Capacity cDNA Reverse Transcription kit (Applied Biosystems, Carlsbad, CA, USA) according to the manufacturer’s instructions. Target gene expression levels were determined using RealHelix™ Premier qPCR kit (NanoHelix, Daejun, Korea) with a StepOnePlus Real-Time PCR System (Applied Biosystems, Carlsbad, CA, USA). Primers for the target genes are listed in Table 2, and the expression level of glyceraldehyde-3-phosphate dehydrogenase (GAPDH) is used as a house-keeping gene. The 2^-ΔΔCT^ method was used to quantify relative mRNA expression levels.

**Table 2:** Oligonucleotide primers used in jejunal qRT-PCR analysis^a^

Gene name	Sequence (forward, reverse)	Reference
*GAPDH*	5′-GAGGGTAGTGAAGGCTGCTG-3’	[14]
	5′-CCACAACACGGTTGCTGTAT-3’	
*CLDN1*	5′-TGGAGGATGACCAGGTGAAGA-3’	[15]
	5′-CGAGCCACTCTGTTGCCATA-3’	
*MUC2*	5′-TTCATGATGCCTGCTCTTGTG-3’	[15]
	5′-CCTGAGCCTTGGTACATTCTTGT-3’	
*IL-6*	5′-GACGAGGAGAAATGCCTGACG-3’	[16]
	5′-CCGAGTCTGGGATGACCACTTC-3’	
*IL-10*	5′-TCTACACAGATGAGGTCCTGCC-3’	[16]
	5′-AGGTGAAGAAGCGGTGACAG-3’	

^a^*Abbreviations*: *CLDN1* claudin-1, *GAPDH* glyceraldehyde-3-phosphate dehydrogenase, *IL-6* interleukin-6, *IL-10* interleukin-10, *MUC2* mucin 2, *OCLD* occludin, *ZO-1* zonula occludens-1

### Statistical analysis

All data were analyzed using analysis of variance (ANOVA) with Statistical System 9.4 (SAS Institute, Cary, NC, USA). Significant differences between treatments were determined using Duncan’s multiple-range tests and were defined at the *P* <  0.05 level.

## Results

### Growth performance

As indicated in Table 3, birds in the BAM and SPL groups showed significantly higher BW and ADG (*P* <  0.05) compared to those in the CON group. There were no significant differences in average feed intake between treatments, however, feed efficiency was significantly higher (*P* < 0.05) in the BAM group compared to CON group.

**Table 3:** Effects of sophorolipid on growth performance of broilers^1,2,3^

Treatment	CON	BAM	SPL	SEM	*P*-value
Initial BW, g	40.16	40.12	40.19	0.045	0.853
Final BW, g	1911.8^b^	2011.6^a^	2010.3^a^	18.587	0.035
ADG, g/d	53.48^b^	56.33^a^	56.29^a^	0.531	0.035
ADFI, g/d	88.62	87.89	90.45	0.719	0.344
FE	0.60^b^	0.64^a^	0.62^ab^	0.014	0.010

^1^Treatments: CON, control group fed with basal diet; BAM, group fed with 10 mg/kg of bambermycin supplemented diet; SPL, group fed with 10 mg/kg of sophorolipid supplemented diet

^2^*Abbreviations*: *ADFI* average daily feed intake, *ADG* average daily gain, *BW* body weight, *FE* feed efficiency, *SEM* standard error of means

^3^A pen is an experimental unit; 6 replications (pens) per treatment; 30 chicks per pen

^a, b^ Mean values within a row have different superscript letters were significantly different (*P <* 0.05)

### Intestinal microbial population

**Fig. 1:**
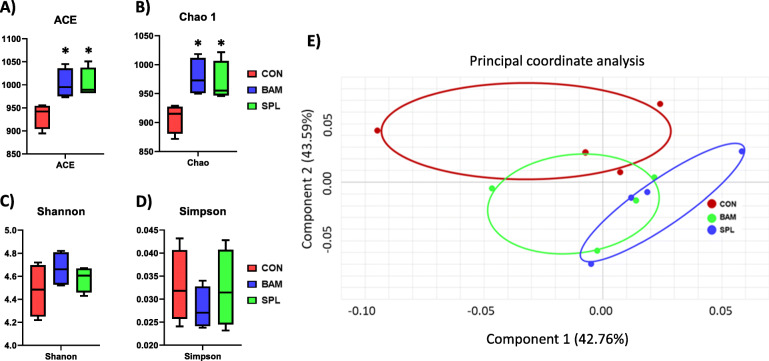
Cecal microbial community of broiler chickens fed experimental diets. **a-b** Dietary effects of bambermycin and sophorolipid on species richness indexes (ACE and Chao1); **c-d** Dietary effects of bambermycin and sophorolipid on diversity indexes (Shannon and Simpson); **e** Principal component Analysis ordination plots of microbial communities in the CON, BAM, and SPL groups based on the Jensen-Shannon distance metric. *P < 0.05 compared with CON group. Treatment groups: CON, control group fed with basal diet; BAM, group fed with 10 mg/kg of bambermycin supplemented diet; SPL, group fed with 10 mg/kg of sophorolipid-supplemented diet

Dietary BAM and SPL supplementation significantly increased ACE and Chao 1 indexes compared to CON group (*P* < 0.05), however, Shannon and Simpson indexes were not different among treatments (Fig. 1a–d). Cecal microbial communities of BAM and SPL groups were partially and completely separated from that of CON group (Fig. 1e).

**Fig. 2:**
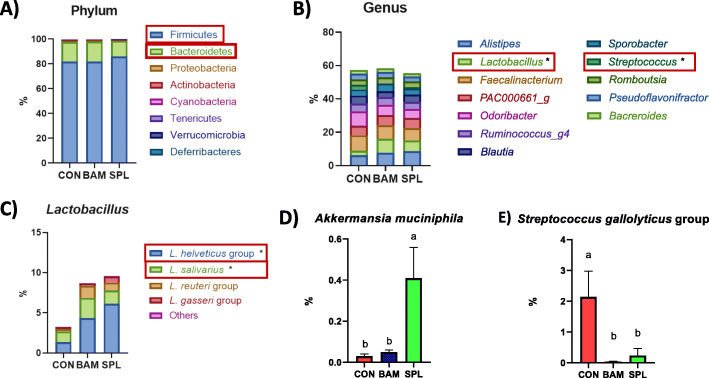
Gut microbiota population of broilers fed with experimental diets. **a** Intestinal microflora at phylum; **b** Intestinal microflora at genus level; **b–d** Specific bacterial populations at the species level in the cecum of birds (*Lactobacillus family, Akkermansia muciniphila,* and *Streptococcus gallolyticus* group). *P < 0.05 compared with CON group. ^a, b^Mean values within a row have different superscript letters were significantly different (*P* < 0.05). Treatment groups: CON, control group fed with basal diet; BAM, group fed with 10 mg/kg of bambermycin supplemented diet; SPL, group fed with 10 mg/kg of sophorolipid-supplemented diet

At phylum level, dietary SPL supplementation increased Firmicutes population and decreased Bacteroidetes population (Fig. 2a). Additionally, dietary SPL supplementation significantly increased (*P* < 0.05) the genus level of *Lactobacillus* and decreased (*P* < 0.05) that of *Streptococcs* (Fig. 2b). At the species level, dietary SPL supplementation significantly increased (*P* < 0.05) the populations of *Lactobacillus helveticus* and *Lactobacillus salivarius* in the SPL group compared to their populations in the CON group (Fig. 2c), and the SPL group also showed a significantly higher (*P* < 0.05) level of *Akkermansia muciniphila* compared to the other treatment groups (Fig. 2d). The population of the *Streptococcus gallolyticus* group was significantly decreased (*P* < 0.05) by dietary BAM and SPL supplementation (Fig. 2e).

### Cecal short-chain fatty acid concentration

As listed in Table 4, dietary SPL supplementation significantly increased (*P* < 0.05) the total concentration of SCFA, and those of acetate and butyrate in the SPL group compared to that in the BAM group. In addition, the ratio of propionate was significantly lowered (*P* < 0.05) in SPL group compared to BAM group.

**Table 4:** Effects of sophorolipid on cecal SCFA concentration of broilers^1,2,3^

Treatment	CON	BAM	SPL	SEM	*P*-value
Absolute concentration of SCFA, μmol/g					
Total SCFA	226.43^a^	179.47^b^	249.61^a^	10.802	0.012
Acetate	185.91^a^	143.14^b^	206.06^a^	9.984	0.017
Propionate	17.62	15.73	17.18	0.676	0.526
*iso*-butyrate	3.69	3.84	4.01	0.208	0.850
Butyrate	19.20^ab^	16.76^b^	22.36^a^	0.978	0.049
Percentage of SCFA, %					
Acetate	82.11	78.98	82.48	0.852	0.187
Propionate	7.76^ab^	8.91^a^	6.88^b^	0.322	0.021
*iso*-butyrate	1.66	2.17	1.60	0.130	0.140
Butyrate	8.47	9.95	9.04	0.615	0.647

^1^Treatments: CON, control group fed with basal diet; BAM, group fed with 10 mg/kg of bambermycin supplemented diet; SPL, group fed with 10 mg/kg of sophorolipid supplemented diet

^2^*Abbreviations*: *SCFA* short-chain fatty acid, *SEM* standard error of means

^3^A chick is an experimental unit; 6 replications per treatment

^a, b^ Mean values within a row have different superscript letters were significantly different (*P* < 0.05)

### Intestinal characteristics and histological analysis

As shown in Table 5, birds fed with the SPL-supplemented diet (SPL group) showed significantly reduced (*P* < 0.05) intestinal weight compared to those of the other treatment groups; however, gut weight per length was not changed by SPL supplementation. Hence, dietary SPL supplementation significantly increased (*P* < 0.05) VH compared to that of birds in the other treatment groups without affecting CD (Table 5). The ratio of VH to CD in the birds fed with the SPL-supplemented diet was the highest (*P* < 0.05) compared to that of birds in CON group. Moreover, the goblet cell numbers per 1 μm of villus were also significantly increased (*P* < 0.05) in the SPL treatment group compared to that in the other groups.

**Table 5:** Effects of sophorolipid on gut characteristic and histological analysis of broilers^1,2,3^

Treatment	CON	BAM	SPL	SEM	*P*-value
Intestinal, g/100 g body weight	3.18^a^	3.08^ab^	2.96^b^	0.037	0.038
Intestinal weight/length, g/m	30.60	31.12	30.54	0.319	0.735
Villus height, μm	371.27^b^	398.17^ab^	422.50^a^	7.483	0.010
Crypt depth, μm	110.58	104.40	105.50	1.366	0.142
Villus height/crypt depth	3.37^b^	3.82^a^	4.01^a^	0.099	0.013
Goblet cells/villus height, /μm	0.22^b^	0.21^b^	0.34^a^	0.016	< 0.001

^1^Treatments: CON, control group fed with basal diet; BAM, group fed with 10 mg/kg of bambermycin supplemented diet; SPL, group fed with 10 mg/kg of sophorolipid supplemented diet

^2^*Abbreviation*: *SEM* standard error of means

^3^A chick is an experimental unit; 6 replications per treatment

^a, b^ Mean values within a row have different superscript letters were significantly different (*P* < 0.05)

### Gene expression levels related to inflammation, tight junction, and mucin secretion

**Fig. 3:**
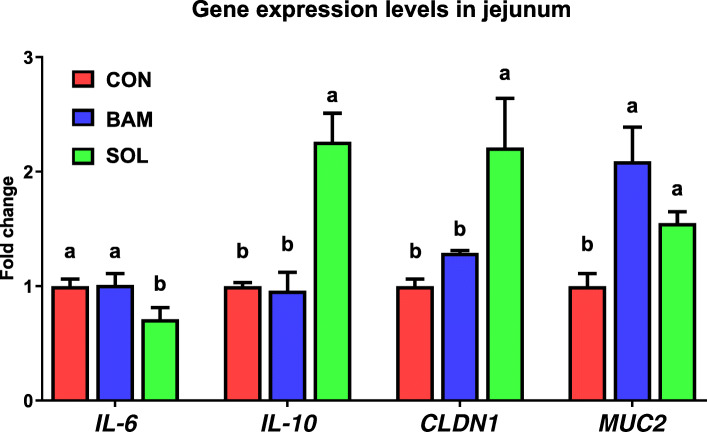
mRNA expression levels of genes related to inflammation, tight junction, and mucin in the jejunum of birds fed with experimental diets. ^a, b^Mean values within a row have different superscript letters were significantly different (*P* < 0.05). Treatment groups: CON, control group fed with basal diet; BAM, group fed with 10 mg/kg of bambermycin supplemented diet; SPL, group fed with 10 mg/kg of sophorolipid-supplemented diet. Abbreviations: *IL-6*, interleukin-6;* IL-10*, interleukin-10; *CLDN1*, claudin-1; *MUC2*, mucin 2

As presented in Fig. 3, dietary SPL supplementation significantly downregulated (*P* < 0.05) the expression level of interleukin-6 (*IL-6*) and upregulated (*P* < 0.05) that of interleukin-10 (*IL-10*) compared to the other treatments. The expression level of claudin-1 (*CLDN1*) significantly increased (*P* < 0.05) in the SPL group compared to the other groups. Moreover, the BAM and SPL groups showed significantly increased (*P* < 0.05) expression level of mucin 2 (*MUC2*) compared to the CON group.

## Discussion

First, the results of this study indicated that SPL could accelerate the growth of broilers as much as bambermycin, which is in agreement with the reports of Boontiam et al. [17] which demonstrated that surfactant supplementation with a low energy and crude protein diet could improve average daily gain and feed efficiency during the overall experimental period without affecting average daily feed intake. Furthermore, both glycolipid-type antibiotics and biosurfactant increased the beneficial microbiota *L. heliveticus* and *L. salivarius* and decreased the pathogenic bacteria *S. gallolyticus*. In accordance with our results, Abu Hafsa and Ibrahim [18] also demonstrated that similar modulation of gut microbiota (increased *Lactobacillus* and decreased *Sterptococcus*) by dietary probiotic supplementation significantly improved the growth of birds. Various reports related *Lacobacillus* species have suggested that *L. heliveticus* is known as the beneficial microbes in bone and mental health and *L. salivarius* has a positive relationship with growth of birds [19–21]. Collectively, microflora communities modulated by bambermycin and SPL were similar, however SPL have seemed to accelerate the shift of the community compared to that of bambermycin. Also, these results suggested that this microbial shift has a potential to improve the growth and health of birds by modulation of their metabolites.

On the other hand, SCFA are the main metabolites of microbial fermentation, and they have been widely studied to elucidate the specific mechanism of antibiotic growth promoter linking the host and its intestinal microbiota [22]. In 2019, Guinan et al. demonstrated that water supply with antibiotic could downregulated the SCFA concentration in cecal contents by increased colonization of *Candida albicans* [23]. In agreement with this study, the results of our study demonstrated that dietary bambermycin supplementation significantly reduced the concentration of total SCFA compared to birds fed control diet and SPL supplemented diet. Additionally, both acetate and butyrate were increased in the SPL group than BAM group, which might be due to the increased populations of butyrate-producing bacteria, *L. helveticus* and mucin-degrading bacteria, *A. muciniphila* [24, 25]. An in vitro study with *L. helveticus* showed that this probiotics strain significantly increased butyrate concentration, and it may be due to the upregulated conversion ratio of lactate to butyrate by lactating-utilizing bacteria [26]. And *A. muciniphila* supplemented with mucin could produce acetate and ethanol from mucin fermentation [27]. Collectively, these results suggest that dietary SPL treatment maintain the cecal SCFA concentration by intestinal colonization of SCFA-producing bacteria unlike bambermycin supplementation.

The gastrointestinal tract is the main region that plays a role in a protective system because it is the first organ to meet external substances with a large contact area [28]. Hence, the gut defense strategy employs various physiological factors (e.g., mucus barrier, tight junctions, and immune cytokines) to maintain the intestinal homeostatic balance [29]. Our results suggest that dietary SPL supplementation could enhance mucin-presenting capacity and villus turnover balance by increasing beneficial bacterial populations. At phylum level, there were higher portion of Firmicutes, and lower that of Bacteroidetes in SPL treatment group compared to the other groups. Bacteroides, known as the LPS-generating bacteria group when they are lysed, may play a critical role to destruct intestinal integrity and morphology by lowering intestinal permeability [30], resulted in the lower Firmicutes/Bacteroidetes ratio is considered as the favorable index of weight loss [31]. At species level, *Akkermansia muciniphila*, has received attention because of its specific biological properties including immune modulation, wound healing, and SCFA production [32]. Moreover, various studies have demonstrated that *A. muciniphila* has the potential to act as a probiotic because it could exert a glucose-lowering effect by regulating gut barrier integrity through increased expression levels of tight junction proteins [33, 34]. Similar to the results of these studies, we also found an increased population of *A. muciniphila* and strengthened gut epithelial integrity and mucus secretion capacity.

Additionally, our results demonstrated that SPL could relieve the local gut immune response and strengthen the intestinal epithelial barrier by modulating the gut microbiota population. *S. gallolyticus*, a gram-positive pathogenic bacterium, which is commonly found in various animals and humans, and it is a potential transmission bacterium with antibiotic resistance [35, 36]. In addition, Li et al. [37] demonstrated that inoculation of *S. gallolyticus* in duckling induced macrophage necroptosis in spleens by bacterial infection and increased the expression levels of* IL-6*, and that decreased population of *S. gallolyticus* achieved in response to dietary oregano powder has a negative relationship with the increase of anti-inflammatory cytokine *IL-10* [38]. In accordance with the results of these previous studies, decreased population of pathogenic bacteria (*S. gallolyticus*) by sophorolipid supplementation has the potential to alleviate immune responses by improving pro- (*IL-6*) and anti-inflammatory (*IL-10*) cytokine production. On the other hands, our results also found that the higher population of *Lactobacillus* genus in BAM and SPL groups compared to CON group. *Lactobacillus* is the genera of gram positive and facultative anaerobes and it can inhibit the pathogen growth by establishment of low pH environment through lactic acid producing capacity [39]. Therefore, our results demonstrated that microbial shift by SPL might be able to modulate immune response in broilers’ intestine.

## Conclusions

Dietary sophorolipid supplementation in broiler feed could modulate the intestinal microbiota population and short-chain fatty acid levels. Hence, the promoted gut environment could improve gut defense integrity, including intensified mucus layer and tight junction, and alleviate local inflammation, resulting in the acceleration of chick growth. Further studies are required to elucidate the precise relationship between sophorolipids and their effects on intestinal health.

## Data Availability

Not applicable.

## Abbreviations

ADFI: Average daily feed intake
ADG: Average daily gain
ANOVA: Analysis of variations
BW: Body weight
CD: Crypt depth
CLDN1: Claudin-1
FE: Feed efficiency
GAPDH: Glyceraldehyde 3-phosphate dehydrogenase
GC***–***MS: Gas chromatography–mass spectrometry
IL-6: Interleukin-6
IL-10: Interleukin-10
MTBSTFA: *N*-tert-butyldimethylsilyl-*N*-methyltrifluoroacetamide
MUC2: Mucin 2
QIIME: Quantitative Insights Into Microbial Ecology
SPL: Sophorolipid
SCFA: Short-chain fatty acid
VH: Villus height
